# Differential localization of HPV16 E6 splice products with E6-associated protein

**DOI:** 10.1186/1743-422X-2-50

**Published:** 2005-06-16

**Authors:** Kulthida Vaeteewoottacharn, Siriphatr Chamutpong, Mathurose Ponglikitmongkol, Peter C Angeletti

**Affiliations:** 1 Department of Biochemistry, Faculty of Science, Mahidol University, Bangkok Thailand; 2 Nebraska Center for Virology, School of Biological Sciences, University of Nebraska-Lincoln, Lincoln, NE 68588, USA

## Abstract

High-risk Human Papillomavirus (HPV) is the etiological agent associated with the majority of anogenital cancers. The primary HPV oncogenes, E6 and E7, undergo a complex splicing program resulting in protein products whose purpose is not fully understood. Previous mouse studies have confirmed the existence of a translated product corresponding to the E6*I splice product. In terms of function, the translated E6*I protein has been shown to bind to E6 protein and to E6 associated protein (E6AP). E6*I has an inhibitory effect on E6-mediated p53 degradation in E6 expressing cells. In order to analyze the relationship between E6*I and full-length E6 in relation to localization, we created a series of green fluorescent protein (GFP) fusion products. The localization of these proteins with reference to E6AP *in vivo *remains unclear. Therefore, we investigated the cellular distribution of different forms of E6 with reference to E6AP. E6 and E6*I proteins, expressed from a wild type E6 gene cassette, were dispersed in the nucleus and the cytoplasm. Whereas, the E6 splice donor mutant (E6MT) was primarily localized to the nucleus. E6*I protein and E6AP were found to co-localize mainly to the cytoplasm, whereas the co-localization of full-length E6 protein and E6AP, if at all, was found mainly at the perinuclear region. These results suggest a functional relationship between the E6*I and full-length E6 protein which correlates with their localization and likely is important in regulation of the E6-E6AP complex.

## Findings

Human papillomavirus (HPV) is a ubiquitous sexually transmitted DNA virus. A subset of mucosal HPVs are termed "high-risk" (for example, types 16, 18 and 31) because of an increased association with cervical cancer (review[1]). Among this group, HPV16 is the most common type, being found in about fifty percent of invasive cancers worldwide [2]. Two HPV16 oncoproteins, E6 and E7, are actively expressed in cervical cancer cells and are responsible for host cell transformation and cancer progression [3,4]. As a polycistronic gene, the transcription of the E6E7 cassette yields E6E7 full-length mRNA as well as two spliced products, E6*IE7 and E6*IIE7. These splicing events are only found in the high-risk HPVs, but not in the low risk group [5]. From previous studies, the E6*I transcript has been found to be the most abundant one detected in HPV16 transformed cells, transgenic animals, cervical cancer cell lines and clinical samples [3,5,6]. While E7 is predicted to translate from spliced products as well as full-length transcripts, E6 protein can only be encoded from full-length transcripts [5,7]. The splicing has been proposed to promote E7 translation by providing space for ribosome initiation to occur [5,8]. However, it has been revealed that the translation of E7 from the full-length transcript is as efficient as those from spliced transcripts and that splicing is not required for E7 synthesis [9].

In earlier studies, difficulty in detection of the truncated E6 proteins raised the possibility that truncated forms of E6 might be present in such diminishingly small quantities that they may not have significant function [10]. However, detection of E6*I and E6*II proteins in HPV18-containing cervical cancer cells established in nude mice [7] and HPV16 transfected cells [11] strengthens the argument that these products have a role in tumorigenesis and in the viral lifecycle. Furthermore, studies of *in vitro *synthesis of E6*I protein from wheat germ extract, but not rabbit reticulocyte lysate, suggest rapid protein turnover in the absence of E6AP [9,12]. To date, various properties of E6*I protein have been studied. For example, HPV16 E6*I protein has the ability to transactivate the adenovirus E2 promoter as well as HPV P97 promoter [13]. The ability of HPV18 E6*I protein to bind to full-length E6 and E6AP (an E3 ubiquitin ligase) has been previously reported [14,15]. The interaction of E6 protein with E6AP was found to be important in E6 functions (review in [16]). However, the information regarding E6 and E6*I protein localization with respect to E6AP protein in the cells is still unclear. Thus, we were interested in analyzing the co-localization of E6 and E6*I protein with E6AP to gain further insight into functional relationships that might correlate with localization.

**Figure 1:**
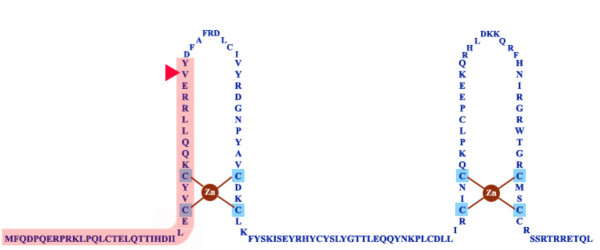
**E6 protein sequences**. The secondary structure of E6 protein is shown. The E6 gene codes for full-length E6 as well as a truncated protein (E6*I, indicated in pink). The E6 splice donor mutant (E6MT) was generated by site-directed mutagenesis at the splice donor site resulting in a V to L substitution at amino acid 42 (arrow head). This construct expresses only E6 full-length protein. Whereas, the E6*I construct expresses only the truncated form of E6 and not the full-length form. GFP was fused to the N-terminus of each of these constructs.

To overcome the difficulty in E6 protein detection by use of antibodies, GFP-linked E6 constructs were created (Figure 1). HPV16 E6 (nucleotide 101 to 559) and E6*I (nucleotide 101 to 417, without nucleotide 226–409) were amplified from SiHa cDNA and cloned in frame to the C-terminus of pEGFP-C1 (Clontech) using *Xho*I and *Kpn*I sites. From previous studies [14,17], similar GFP-E6 constructs have been shown to generate both full-length E6 as well as E6*I proteins. The E6 splice donor mutant (E6MT) was generated by site-directed mutagenesis at the spliced donor site with a G to C mutation at nucleotide 226 resulting in a V to L amino acid substitution. Plasmids were transfected into human embryonic kidney cells, 293T, and immortalized human keratinocyte cells, HaCaT, using FuGene 6 transfection reagent (Roche) using methods prescribed by the manufacturer. At 48 hours after transfection, cells were fixed with 4% paraformaldehyde in PBS, permeabolized with 0.1–0.5% NP-40, blocked with 5% fetal bovine serum in PBS and incubated with monoclonal antibody against E6AP (Sigma). After three washes, cells were incubated with Alexa 568-conjugated to goat anti-mouse antibody (Molecular Probes). Cells were washed with PBS and mounted on the slides using Gel Mount (Sigma). To localize the nuclei, cells were incubated with 4',6-Diamidino-2-phenylindole (DAPI; Roche) diluted in PBS before a final PBS washing step. Slides were observed using an Olympus FV500 Confocal microscope with 3 lasers giving excitation lines at 405 nm, 488 nm and 515 nm. The images were taken under 60 × or 100 × objective oil immersion lens and collected at 1,024 × 1,024 pixels or 512 × 512 pixels resolution. The images were captured at various scanning conditions suited for the individual samples in order to eliminate overlapping signals between channels.

**Figure 2:**
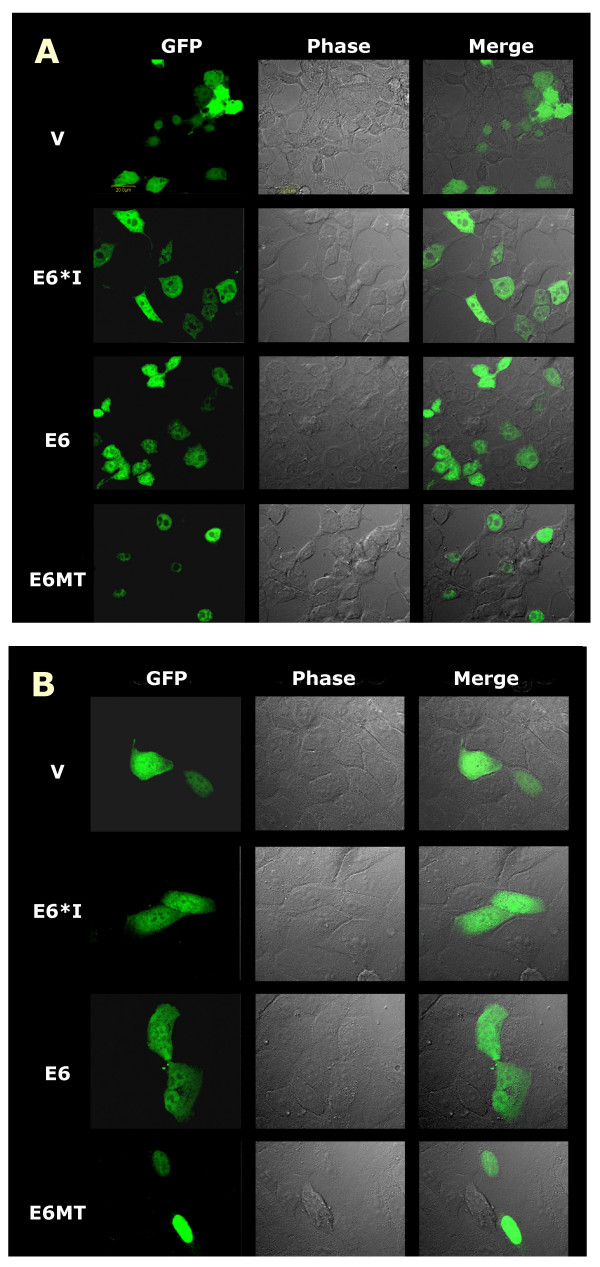
**Cellular localization of HPV16 E6s in 293T (A) and HaCaT (B)**. Cells were transiently transfected with plasmids expressing GFP, GFP-E6, GFP-E6*I and GFP-E6MT. Cells were grown and fixed with 4% paraformaldehyde in PBS on the coverslips at 48 h after transfection. The fluorescent images, phase contrast images and merge fluorescent-phase contrast images are shown. V indicates the pEGFP-C1 vector transfected cells. The scale bar represents 20 μm.

We observed the localization of the different forms of E6 protein in 293T cells and HaCaT cells. The results are shown in figure 2. The wildtype E6 construct, which yields both full-length E6 and E6*I proteins (the truncated form of E6 that contains 43 amino acids identical to the N-terminus of E6), was expressed throughout the cells, but preferentially in the nucleus. A similar result was obtained with E6*I transfected cells. Interestingly, when E6MT was expressed, the signal was predominantly detected in the nucleus. All three protein products were excluded the nucleoli. Similar results were obtained from both 293T and HaCaT cells. These results correspond to previous reports of HPV18 E6 localization [17,18]. However, the localizations of E6 and E6*I proteins are different than one previous study of HPV16 E6 [19] which found that HPV16 E6 and E6*I proteins were primarily nuclear. Since HPV16 E6 used in our study was amplified from SiHa cells, which contains two mutations (position 83, L to V and position 103, Q to D), it is possible that these contribute to the observed differences. These conservative amino acid changes are not predicted to alter localization since neither are in regions responsible for nuclear import [19]. However, alteration of interactions with unknown cellular targets by these mutations cannot be ruled out. In our experiments, the level of E6*I and/or E6 proteins differed slightly from the other studies. Though the signals shown in our experiments from E6 and E6*I proteins were dispersed throughout the cells, these signals were consistently excluded from nucleoli. Hence, these signals are not likely to be artifactual.

**Figure 3:**
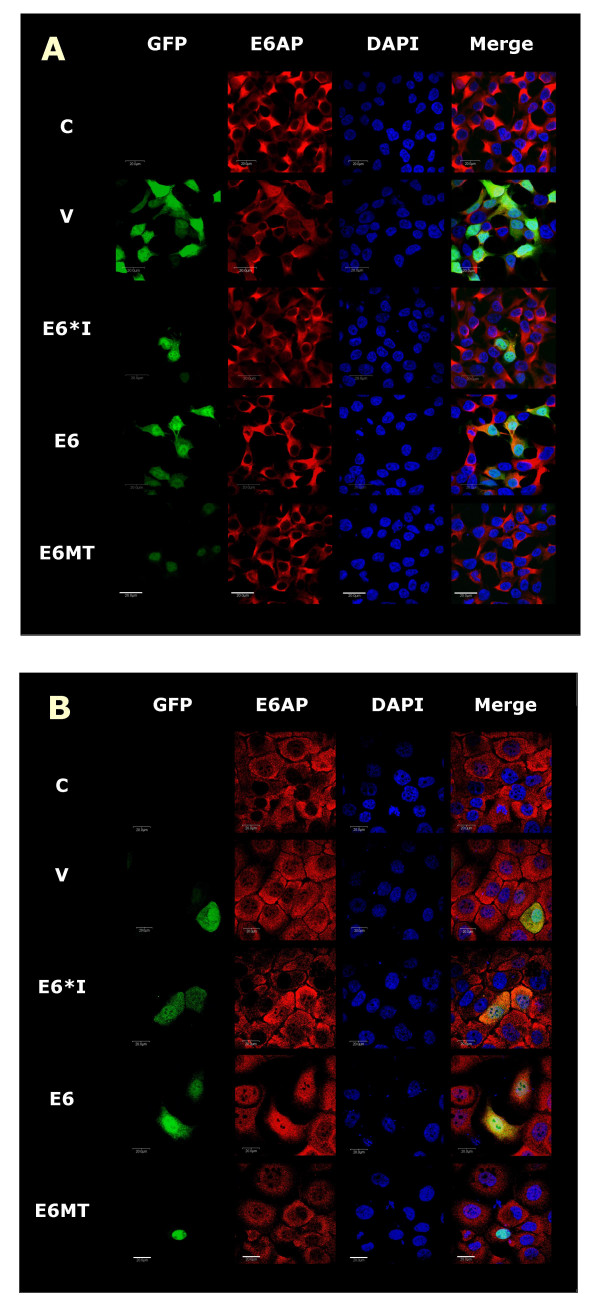
**Co-localization of E6s and E6AP in 293T (A) and HaCaT (B)**. Transiently transfected cells were analyzed for E6 or E6 variants fused to GFP, E6AP (Alexa 568 dye) and nuclear DNA (DAPI) by confocal microscopy. Slides were analyzed by microscopy with 3 lasers excitation lines. The images from the individual channels (DAPI, GFP, Alexa 568) as well as the merged image are shown. P and V represent non-transfected cells and pEGFP-C1 vector transfected cells, respectively. The scale bar represents 20 μm.

As the E6 protein has been shown to associate with E6AP in HPV-induced cervical cancers, the location of these functional complexes is of importance. From results in figure 3, co-localization of either E6 or E6*I protein and E6AP mainly occured in the cytoplasm whereas co-localization of E6MT and E6AP was restricted to areas of the nucleus and the perinuclear region. In essence, E6MT protein was rarely co-localized with E6AP. The localization of E6AP agreed with the previous studies showing that E6AP is predominately localized to the cytoplasm, and to a lesser extent, the nucleus[20,21]. E6 protein expression has been shown to induce self-ubiquitination and degradation of E6AP with a dramatic reduction in E6AP half-life overall [22]. This likely explains the infrequent detection of E6MT and E6AP co-localization in the cytoplasm. The binding of E6AP with full-length E6 protein leads to the rapid degradation of E6AP. The detection of E6*I protein co-localized with E6AP is in agreement with previous observations which showed interaction between HPV18 E6*I protein and E6AP *in vitro *[14,15] and that we would expect such complexes to be stable since E6*I inhibits E6AP function thereby allowing the complexes to be visible by immunofluoresence. The co-localization of E6 protein with E6AP resembles that of E6*I. The presence of translation products of E6*I protein in E6-transfected cells, may explain this similarity. There are two mechanistic possibilities; first, the E6*I protein may compete with E6 protein in binding to E6AP and this binding may protect E6AP from degradation induced by E6 protein. Second, the E6*I protein might bind to E6 full-length protein and the binding might modulate the function of E6 in induction of E6AP degradation. As shown in the microscopy results, the co-localization of E6AP is not exclusively to E6 protein. This raises the possibility that E6AP-independent functions of E6 [23], E6AP-independent degradation of E6 [24] or, in fact, E6-independent functions of E6AP may play a significant role.

In addition, we observed stronger fluorescent signals with E6-transfected cells than from E6*I or E6MT-transfected cells (data not shown). More intense signals might suggest interaction with and stabilization by cellular proteins. This could involve the ability of E6 protein to oligomerize with E6 or E6*I protein [14]. In other studies, binding of E6*I and E6 protein has been shown to induce the degradation of both proteins [14], however, the increase in signal observed with E6 suggests that homo-oligomeric interactions could stabilize the protein complexes. The differing capacity of E6*I protein of different HPV types to interfere with E6 might result in differing downstream effects on E6-dependent targets. Since the similarity of E6*I protein between HPV16 and HPV18 is about 50% and the amino acids 28–31, which have been shown to be important in E6 and E6AP binding, differ slightly (amino acid "IEIT" from HPV18 versus "IILE" from HPV16), this may account for the differing capacity to bind E6 or E6AP. Furthermore, HPV16 E6 and HPV18 E6 have been shown to have different preferences in binding to p53 structural conformations [20]. Therefore, it might be informative to test E6*I protein from various HPV types for functional differences in their abilities to affect stability of cellular targets such as p53.

**Figure 4:**
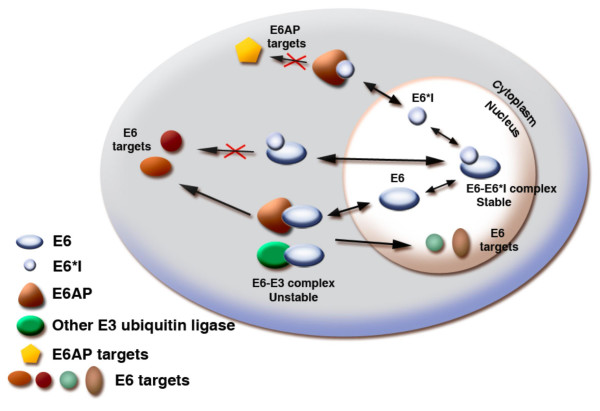
A model for regulation of E6 protein function by E6*I. E6*I protein binds to E6 or E6AP in the nucleus and cytoplasm. The binding may lead to the inhibition of E6-E6AP binding as well as inhibition of binding of E6 or E6AP to other cellular targets. In this manner, E6*I may inhibit either E6-induced or E6AP-dependent proteosome degradation function or, in fact, inhibit other functions of either E6 or E6AP. The question mark (?), represents other E3 ubiquitin ligases enzymes participating in E6-mediated degradation or E6AP-independent, E6-induced protein degradation.

In conclusion, our results suggest a functional role for expression of E6*I protein in high-risk HPV-infected cancer cells. We propose a model as shown in figure 4. E6*I protein may bind to either E6 or E6AP and binding may modulate their functions or interfere directly with degradation of these two proteins. Further studies in the regulation of E6 protein with E6*I protein provide useful insights into HPV-disease and a potential means to control development and progression of HPV-related cancers.

## List of abbreviations

Human papillomavirus (HPV), E6-associated protein (E6AP), 4',6-Diamidino-2-phenylindole (DAPI), green fluorescent protein (GFP)

## Competing interests

The author(s) declare that they have no competing interests.

## Authors' contributions

K. V. designed and created the GFP fusion constructs, performed all of the described experiments and interpreted the data. S. C. created the E6MT mutant. P. M. and P. C. A. provided logistical, financial and material support and helped in data interpretation and manuscript preparation.
